# Designing and modeling of a novel electrolysis reactor using porous cathode to produce H_2_O_2_ as an oxidant

**DOI:** 10.1016/j.mex.2019.05.036

**Published:** 2019-06-01

**Authors:** Reza Ali Fallahzadeh, Mohammad Hassan Ehrampoush, Amir Hossein Mahvi, Mohammad Taghi Ghaneian, Arash Dalvand, Mohammad Hossein Salmani, Hossien Fallahzadeh, Mohsen Nabi Meybodi

**Affiliations:** a Environmental Science and Technology Research Center, Department of Environmental Health Engineering, Shahid Sadoughi University of Medical Sciences, Yazd, Iran; b Department of Environmental Health Engineering, School of Public Health, Tehran University of Medical Sciences, Tehran, Iran; c Center for Solid Waste Research, Institute for Environmental Research, Tehran University of Medical Sciences, Tehran, Iran; d Department of Biostatistics and Epidemiology, School of Health, Shahid Sadoughi University of Medical Sciences, Yazd, Iran; e Department of Pharmaceutics, Faculty of Pharmacy, Shahid Sadoughi University of Medical Sciences, Yazd, Iran

**Keywords:** Advanced Oxidation Process (AOP), Electro-oxidation (EO), Electrochemical Oxidation Processes (EOP), Box Behnken design, Electro-oxidation process, Hydrogen peroxide, Modeling, Porous electrode

## Abstract

The entry of toxic organic pollutants and resistant to biodegradation has increased the concern about human health. The use of advanced oxidation (AO) processes to degrade these pollutants has been developing. One of the AO processes is based on the use of hydrogen peroxide in removing resistant organic pollutants.

This study aimed to develop a new reactor capable of producing H_2_O_2_ in the solution. Therefore, a porous electrode made of stainless steel with the capability of air injection in the electrode center was used. The 30 cm rod graphite electrodes were also used as an anode electrode in a 4000 ml reactor. The effects of variables, including current density (30–40 mA/cm^2^), time (10–30 min), and electrolyte concentration (12–17 mM/L) on the amount of H_2_O_2_ production were evaluated by *Box behenken* design under response surface methodology using *Design expert* software. The results of this study showed that H_2_O_2_ can be produced at the electrode surface of porous cathode under optimal conditions of 36 mA/cm^2^ current density, 16 mM/l electrolyte concentration, in 23 min, and in the amount of 34 ppm. Using a porous cathode electrode causes the maximum contact among the solution, water, and air, and increases the production of H_2_O_2_.

The release of resistant organic compounds to the waste water is a serious problem to the environment. By the application of the *Electro-oxidation* (EO)reactor with the ability to produce H_2_O_2_, this issue is resolved. Furthermore, this technique is applied for non-selective degradation of the toxic organic compounds.

• The electro-oxidation process is a useful method for destruction of persistent organic matter from wastewater.
• Due to use of porous cathode in this method, contact between the electrode and the sewage is at its maximum level which increases the efficiency and speed of sewage treatment.
• This method can produce H_2_O_2_ as a high potential oxidant that can reduce persistent organic properties of sewage and make the wastewater suitable for biological treatment.

## Graphical abstract

**Figure:**
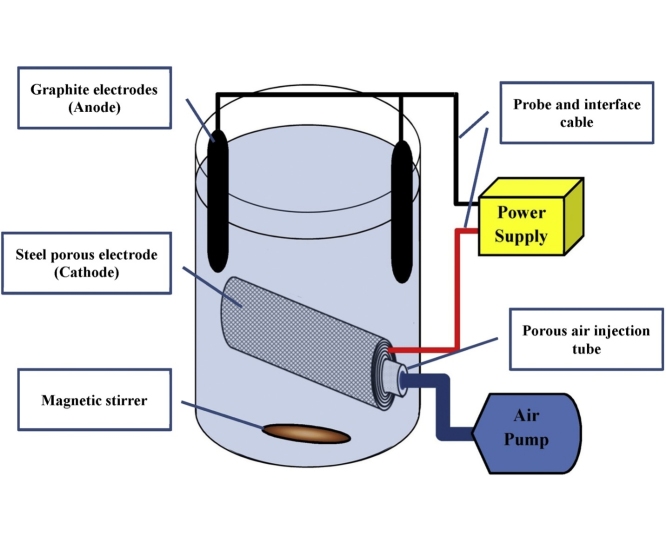

**Specifications Table**

**Table:** 

Subject Area:	• *Chemistry* • *Environmental Science*
More specific subject area:	*Treatment of water and wastewater*
Method name:	*Advanced Oxidation Process (AOP), Electro-oxidation (EO), Electrochemical Oxidation Processes (EOP)*
Name and reference of original method:	• *Advanced Oxidation Process (AOP)* [1] • *Electro-oxidation (EO)* [2] • *Electrochemical Oxidation Processes (EOP)* [3,4]
Resource availability:	• *DC power supply (DAZHENG PS-305D, China)* • *Air pump (2 l/min)* • *Stainless steel netting, wire mesh screen, 0.21 mm* • *Graphite rod electrodes (8 × 300 mm)* • *Magnet mixer (150 rpm)* • *Design-Expert 10.0 Software*

## Method details

Recently, human societies development and the use of various compounds have led to the presence of micro-pollutants in the environment, which, in addition to the difficult detection of contaminants, are sometimes resistant to bio-degradation [[5], [6], [7], [8], [9]]. On the other hand, using biological processes based on genetically modified bacteria and algae to eliminate toxic compounds is associated with challenges [[10], [11], [12]]. Therefore, the use of physicochemical methods in removing resistant pollutants has been accepted [13,14]. The use of peroxide is an AO process for the removal of resistant pollutants from the environment [15].

The EO process as a simple, economical, safe, and eco-friendly technology is capable of degrading compounds that are resistant to biodegradation by anodic direct and indirect oxidation [16,17]. Indirect oxidation in the EO process is due to the oxidizing compounds produced by the EO process, and these compounds can non-selectively oxidize organic compounds [18,19]. H_2_O_2_ is used as an oxidant in oxidation processes and as a propellant for hydroxyl radical production in Fenton processes [20]. This compound can be produced at the cathode surface by the correct design of the reactor and it can oxidize organic compounds.

One of the major parameters affecting the type and amount of produced oxidants is the shape and type of electrode [21]. Using stable electrodes reduces costs; moreover, increasing the electrode surface increases the level of wastewater contact with electrodes, which results in more organic compounds degradation [22]. In order to increase the contact between the electrode and wastewater, the use of porous electrodes has been developed in wastewater treatment industry [23]. Furthermore, by changing the type of cathode electrode, in addition to increasing the active surface, it is possible to improve oxidizing compounds production, which is the purpose of designing and constructing the modified electrolysis reactor in this study.

**Fig. 1:**
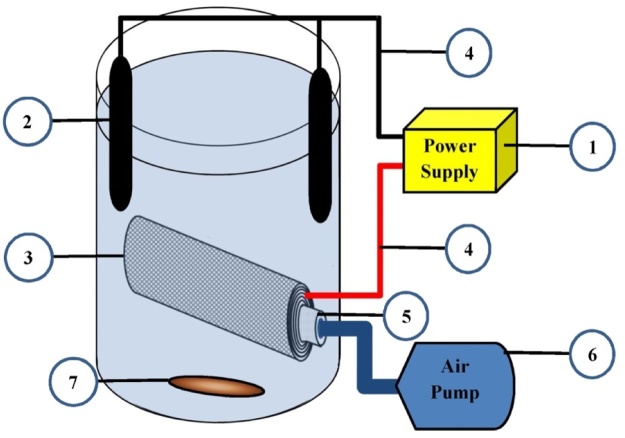
The schematic of the reactor used in the study.

Referring to Fig. 1 and the explanations provided, a better understanding of the EO reactor can be achieved. The components in Fig. 1 do not necessarily require scaling, and instead, the principles of the reactor are emphasized.

Fig. 1 is a transverse perspective view of the reactor and its components. The technical map provided by various components of the system is presented as follows. In this map, the referenced numbers indicate the relevant parts in the technical map.

This reactor includes -1- an electrical current supply connected to -2- the rod graphite electrodes as an anode electrode and -3- a steel porous electrode as a cathode electrode by -4- the probe and interface cable. In the center of the steel porous electrode, there is -5- a porous air injection tube. The air is injected through -6- a blower pump to the cathode electrode center, which affects the H_2_O_2_ production by the electrolysis process. The reactor contents are mixed by -7- a magnetic stirrer.

In this reactor, H_2_O_2_ can be produced by reducing 2 electrons from oxygen (either purely or by injecting air into the solution) at the cathode surface under acidic or normal conditions (with Eº = 0. 68 V/SHE) according to Eq. (1) [24].

(1) $${{\text{O}}_{2}}_{\left(g\right)}+2{\text{H}}^{+}+2e^{-}\to {\text{H}}_{2}{\text{O}}_{2}$$

Eq. (1) is simpler than reducing 4 electrons from water oxygen with Eº = 1. 23 V/SHE [25]. The advanced oxidation (AO) process performed by the oxidants produced by the electrochemical process is called anodic oxidation with electro-generated H_2_O_2_ [26]. In order to maintain the H_2_O_2_ efficiency produced by the electrochemical method, oxygen, cathode, and water contact must be maintained at its maximum value. Therefore, using porous cathodes having a high specific surface is preferable to produce higher amounts of H_2_O_2_ [27]. In this study, porous stainless steel was used as a cathode electrode. Porosity increases the contact between the electrode and the water. The aeration process was also used to increase the oxygen contact with water and the electrode by injecting air into the cathode electrode center. Using porous electrode and air injection into the electrode center maintains oxygen, cathode, and water contact at its maximum, resulting in high H_2_O_2_ production.

## Case study

The mentioned process was evaluated within a reactor with the following characteristics. The amount of produced H_2_O_2_ was determined by *Iodometric* method and titration with sodium thiosulfate in accordance with Eqs. (2)–(5). Finally, according to the amount of thiosulfate consumed, it was measured by Eq. (6).

(2) $${\text{H}}_{2}{\text{O}}_{2}\to {\text{O}}_{2}+2{\;\text{H}}^{+}+2\;{\text{e}}^{-}$$

(3) $$2\;{\text{I}}^{-}\to {\text{I}}_{2}+2\;{\text{e}}^{-}$$

(4) $$2\;\text{N}{\text{a}}_{2}{\text{S}}_{2}{\text{O}}_{3}+{\text{I}}_{2}\to \text{N}{\text{a}}_{2}{\text{S}}_{4}{\text{O}}_{6}+2\text{N}\text{a}\text{I}$$

(5) $$2\;\text{K}\text{I}+{\text{H}}_{2}\text{S}{\text{O}}_{4}+{\text{H}}_{2}{\text{O}}_{2}\to {\text{K}}_{2}\text{S}{\text{O}}_{4}+2\;{\text{H}}_{2}\text{O}+{\text{I}}_{2}$$

(6) $${\text{M}}_{{\text{H}}_{2}{\text{O}}_{2}}=\;\text{m}{\text{l}}_{{\text{N}\text{a}}_{2}{\text{S}}_{2}{\text{O}}_{3}}\times \frac{\;\text{m}\text{o}{\text{l}}_{{\text{N}\text{a}}_{2}{\text{S}}_{2}{\text{O}}_{3}}}{{\text{L}}_{{\text{N}\text{a}}_{2}{\text{S}}_{2}{\text{O}}_{3}}}\times \frac{{\text{m}\text{o}\text{l}}_{{\text{I}}_{2}}}{\;\text{m}\text{o}{\text{l}}_{{\text{N}\text{a}}_{2}{\text{S}}_{2}{\text{O}}_{3}}}\times \frac{{\text{m}\text{o}\text{l}}_{{{\text{H}}_{2}\text{O}}_{2}}}{{\text{m}\text{o}\text{l}}_{{\text{I}}_{2}}}\times \frac{1}{10\;\text{m}\text{l}}$$

The reactor used in this study was a 4000 ml batch reactor. In this reactor, two rod graphite electrodes of 30 cm in length and 0.8 cm in diameter as an anode electrode and a porous stainless steel electrode as a cathode electrode with an internal porous tube for air injection were placed. The input electrical current of the reactor was controlled by the *DAZHENGPS-305D* power supply. The mixing process was performed by a magnetic stirrer at a constant speed of 150 rpm. An aeration diffuser was used to inject 2 liters/min air into the center of porous steel electrode. *Merck* Company NaCl from *Germany* was used as electrolyte.

The amount of H_2_O_2_ production was evaluated in the reactor at different levels of electrolyte concentrations, current intensity, and residence time (Table 1). Therefore, the number of experiments and optimal conditions for the three variables, including residence time, electrolyte concentration, and current density was determined using the *Design Expert* software (version 10) and *Box Behnken* Design. The range for each of the variables was performed at three levels. In order to analyze the experimental results, the response surface regression was used and optimal conditions for the H_2_O_2_ production were presented by the *Polynomial second order model* according to Eq. (7) [28].

(7) $$\text{Y}={\text{β}}_{0}+\sum_{\text{i}=1}^{\text{k}}{\text{β}}_{\text{i}}{\text{X}}_{\text{i}}+\sum_{\text{i}=1}^{\text{k}}{\text{β}}_{\text{i}\text{i}}{\text{X}}_{\text{i}}^{2}+\sum_{\text{i}=1}^{\text{k}}\sum_{\text{j}=1}^{\text{k}}{\text{β}}_{\text{i}\text{i}}{\text{X}}_{\text{i}}{\text{X}}_{\text{j}}+\text{ε}$$

In this equation, *Y* is the amount of produced H_2_O_2_, *Xi* and *Xj* are coded variables, *β0* is the model constant coefficient, *βi*, *βii*, and *βij* are linear, quadratic, and interaction coefficients, respectively.

**Table 1:** Variables and levels considered in accordance with Box Behnken design.

Level			
Upper	Central	Lower	Variable
17	14.5	12	Electrolyte concentration (mM/L)
40	35	30	Current density (mA/cm^2^)
30	20	10	Retention time (min)

## Results and discussion

Based on the *Box Behnken* design, 17 runs was determined to examine the effect of the three variables, including residence time, electrolyte concentration, and current density.

**Fig. 2:**
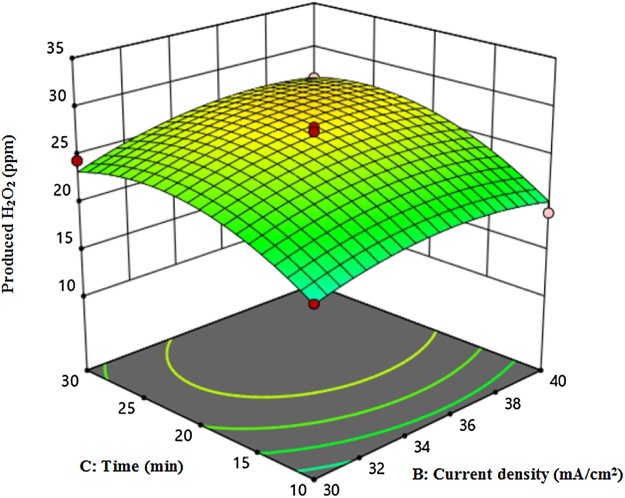
The amount of H_2_O_2_ production based on current density and time.

**Fig. 3:**
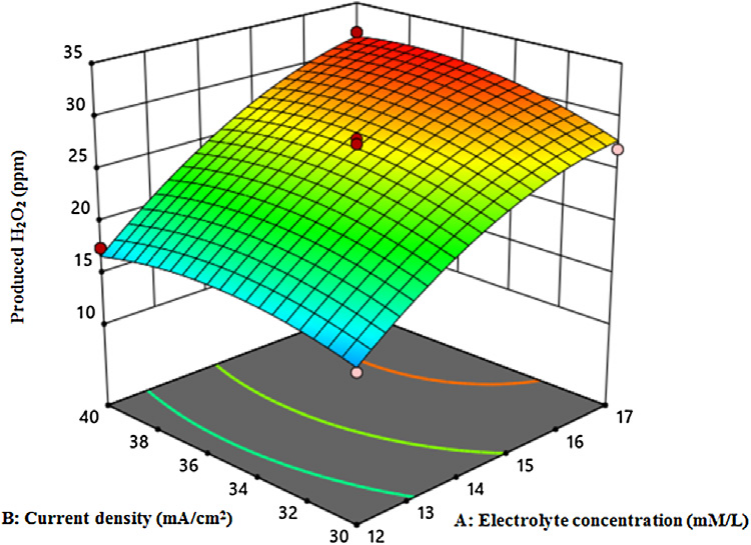
The amount of H_2_O_2_ production based on current density and electrolyte concentration.

**Fig. 4:**
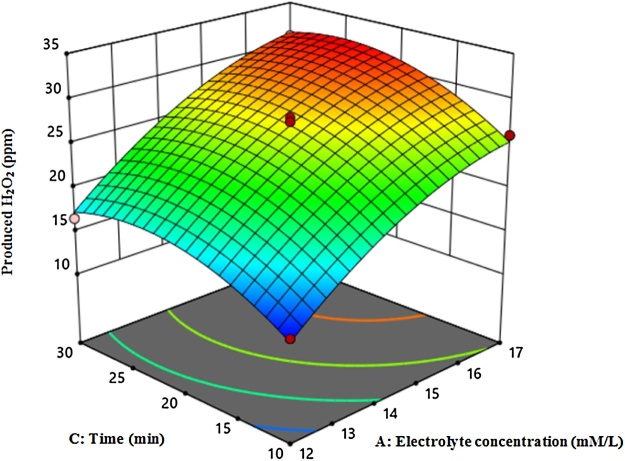
The amount of H_2_O_2_ production based on time and electrolyte concentration.

Fig. 2, Fig. 3, Fig. 4 shows the amount of H_2_O_2_ production based on current density and time, current density and electrolyte concentration, and time and electrolyte concentration, respectively. *ANOVA* analysis based on Quadratic Model showed that lack of fit was not significant. Moreover, the obtained values for R-Squared and Adj R-Squared were 0.98 and 0.95, respectively. In Eq. (8), the amount of H_2_O_2_ production and regression coefficients are presented.

(8) $$\text{P}\text{r}\text{o}\text{d}\text{u}\text{c}\text{e}\text{d}\;{\text{H}}_{2}{\text{O}}_{2}=+27.\;10+6.\;69\times \text{A}+1.\;13\times \text{B}+2.\;69\times \text{C}+0.\;75\times \text{A}\times \text{B}+0.\;375\times \text{A}\times \text{C}+0.\;5\times \text{B}\times \text{C}-2.\;36\times {\text{A}}^{2}-1.\;74\times {\text{B}}^{2}-3.\;11\times {\text{C}}^{2}$$

According to Eq. (8), electrolyte concentration (A), current density (B) and time (C) coefficients are positive, indicating that these variables are directly related to H_2_O_2_ production.

**Fig. 5:**
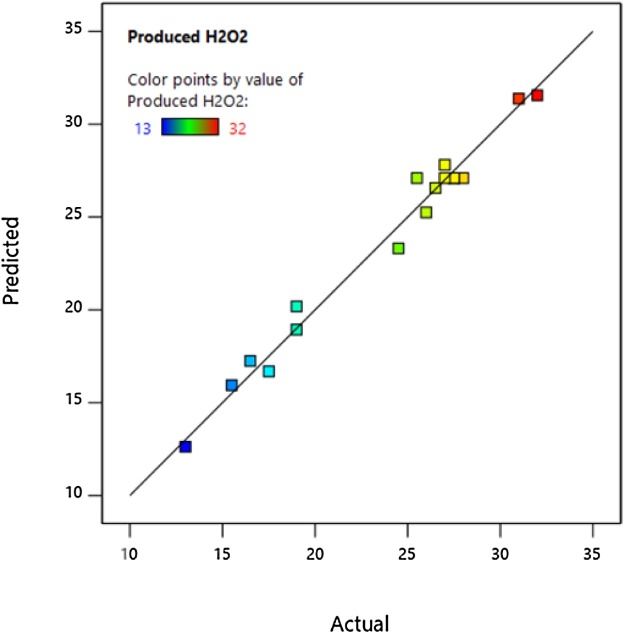
Actual and predicted H_2_O_2_ production.

The amount of real H_2_O_2_ production and the predicted amount by the model, as well as the optimal predicted conditions, are presented in Table 2. Fig. 5 shows the actual and predicted amount of H_2_O_2_ production.

**Table 2:** Optimal conditions for the three variables (current density, time, and electrolyte concentration).

Parameter	Current density (mA/cm^2^)	Time (min)	Electrolyte concentration (mM/L)	H_2_O_2_ Producing (ppm)	
				Predicted	Experimental
Optimum condition	*36*	*23*	*16*	*32.3*	*34*

Based on the results, production of hydrogen peroxide at the desired level by the designed reactor enables the reactor to produce hydrogen peroxide in aquatic environments and degrade organic compounds resistant to bio-degradation. Many studies have emphasized the use of peroxides in removing resistant organic compounds [15,29].

## Concluding remarks

Developing a reactor that is capable of producing H_2_O_2_ as a potential oxidizing agent in the aquatic environment through the electrochemical process was the main aim of this study. The results of this study showed that the use of porous stainless steel cathode as well as air injection to its center, causes the maximum contact surface of the electrode, air, and solution which produces significant amounts of H_2_O_2_.

## Conflict of interest

The authors declare that they have no conflict of interests.
